# Screening for Child Sexual Exploitation in Online Sexual Health Services: An Exploratory Study of Expert Views

**DOI:** 10.2196/jmir.5911

**Published:** 2017-02-14

**Authors:** Victoria Spencer-Hughes, Jonathan Syred, Alison Allison, Gillian Holdsworth, Paula Baraitser

**Affiliations:** ^1^ King's College London London United Kingdom; ^2^ Independent Researcher London United Kingdom; ^3^ Lambeth and Southwark Public Health Directorate London United Kingdom; ^4^ King's College Hospital NHS Foundation Trust London United Kingdom

**Keywords:** Internet, child abuse, sexual, adolescent health services, sexually transmitted diseases, risk assessment

## Abstract

**Background:**

Sexual health services routinely screen for child sexual exploitation (CSE). Although sexual health services are increasingly provided online, there has been no research on the translation of the safeguarding function to online services. We studied expert practitioner views on safeguarding in this context.

**Objective:**

The aim was to document expert practitioner views on safeguarding in the context of an online sexual health service.

**Methods:**

We conducted semistructured interviews with lead professionals purposively sampled from local, regional, or national organizations with a direct influence over CSE protocols, child protection policies, and sexual health services. Interviews were analyzed by three researchers using a matrix-based analytic method.

**Results:**

Our respondents described two different approaches to safeguarding. The “information-providing” approach considers that young people experiencing CSE will ask for help when they are ready from someone they trust. The primary function of the service is to provide information, provoke reflection, generate trust, and respond reliably to disclosure. The approach values online services as an anonymous space to test out disclosure without commitment. The “information-gathering” approach considers that young people may withhold information about exploitation. Therefore, services should seek out information to assess risk and initiate disclosure. This approach values face-to-face opportunities for individualized questioning and immediate referral.

**Conclusions:**

The information-providing approach is associated with confidential telephone support lines and the information-gathering approach with clinical services. The approach adopted online will depend on ethos and the range of services provided. Effective transition from online to clinic services after disclosure is an essential element of this process and further research is needed to understand and support this transition.

## Introduction

Safeguarding children is “the action we take to promote the welfare of children and to protect them from harm” [1]. One form of harm is child sexual exploitation (CSE), which involves those younger than 18 years in exploitative situations, contexts, and relationships in which they receive something (eg, gifts or money) for engaging in sexual activity [2]. Determining the incidence of CSE is complex and underreporting is common [3], but it is estimated that there were 16,500 children at risk in England during the period from April 2010 to March 2011 [4].

Young people experiencing sexual exploitation may use sexual health services even when they have disengaged with other statutory services [2]. Therefore, sexual health services have an important role in identifying CSE. They fulfill this role through routine history taking based on national guidelines for users younger than age 18 and clearly specified referral pathways when concerns are identified [2]. In England, the age of sexual consent is 16 years and all those younger than 18 years are considered at risk of CSE.

Sexual health services are increasingly provided online [5]. The online interface is particularly attractive to young people who value the accessibility, convenience, and discretion of online services [6,7]. Online sexual health services come in many forms from those that only provide sexually transmitted infection tests to comprehensive provision of testing, treatment, and contraception in association with text, telephone, and webchat support and referral to clinical services. The common element of these services is the lack of face-to-face contact, but they may offer different levels of clinical support and links to other relevant services. The lack of face-to-face contact raises specific concerns about processes for identification of risk of CSE. Some of the signs of risk for CSE are difficult to assess online, such as poor self-care, injuries, emotional symptoms, trauma symptoms, or self-harming behavior [4]. At present, online services are limited to those older than 16 years.

There is little national or international guidance on safeguarding within an online sexual health service. A literature search using the terms (or variations of) safeguard, online, Internet, and Web on the databases Allied and Complementary Medicine Database (AMED), British Nursing Index (BNI), Cumulative Index to Nursing and Allied Health Literature (CINAHL), Embase, Health Business Elite, Health Management Information Consortium (HMIC), MEDLINE, and PsychINFO found no directly relevant information to guide policy development.

Due to the lack of published evidence to inform policy development in this area, we interviewed local and national expert practitioners in safeguarding and sexual health to document their views on safeguarding young people using online sexual health services.

## Methods

### Ethical Approval

Ethical approval was received from King’s College London Ethics Committee (REC Reference Number *:* BDM/13/14-102).

### Study Design

Semistructured interviews were completed with expert practitioner stakeholders. Following national guidance on involving the potential beneficiaries of research in research design, data collection, and analysis [8], we worked with young people from a local government-funded school as cointerviewers. Participation was voluntary and open to all students aged 16 to 18 years living in the local area who wanted to be involved in health services research that affected young people. The cointerviewers developed four questions for the interview schedule through a collaborative process led by two of the researchers (JS and AA). One young person attended each of the interviews, where possible, to ask these questions.

### Sampling and Recruitment

A purposive sample of local and national practitioner experts on safeguarding, young people’s rights, and sexual health services was recruited. We identified possible respondents using a snowballing technique in which local experts identified relevant organizations and then purposively sampled from this group for maximum variability to include a wide range of organizations that had direct influence over CSE protocols and child protection policies (Table 1). All statutory and National Health Service organizations in the geographical area were included. Participants chose to give their views either on behalf of their named organization or in a personal capacity.

**Table 1:** Organizations and professional roles of interviewees

Organization	Role	Regional/National
Brook: national sexual health charity for young people	Chief executive	National
English National Chlamydia Screening Programme	Director	National
English National Chlamydia Screening Programme	Quality assurance manager	National
Children’s charity offering support and protection to children being abused	Message board manager speaking in personal capacity	National
Local statutory organizations with responsibility for safeguarding children	Chair	Regional
Local statutory organizations with responsibility for safeguarding children	Development manager	Regional
Local statutory organizations with responsibility for safeguarding children	Director of children’s social care	Regional
Local statutory organizations with responsibility for safeguarding children	Public health consultant	Regional
Local statutory organizations with responsibility for safeguarding children	Senior manager service development	Regional
Hospital services provider	Sexual health promotion manager	Local
Community sexual health services provider	Safeguarding lead clinician	Local
Sexual assault referral center	Senior clinician	Regional
Regional health care commissioning body	Safeguarding nurse	Regional
Youth/housing services	Safeguarding manager	Local

### Data Collection

A total of 14 interviews, lasting 40 to 60 minutes, were completed at the researcher’s or the participants’ place of work with a young person researcher present in eight of them. The interviews were recorded, transcribed, and then analyzed in Nvivo10 (NVivo qualitative data analysis Software; QSR International Pty Ltd, version 10, 2012).

The interview included four sections: experience of safeguarding in clinic and online services, differences between the two safeguarding environments, and the scenario presented in Textbox 1.

**Scenario for interview.**

Apple is 16 years old and has been having sex. She does not feel able to go to her family GP or a local clinic to get an STI test but would like to check that she is healthy. Apple goes online to the online sexual health service website, completes the online risk assessment [which asks questions about the same issues as clinics do].

The discussion was repeated with Apple aged 15 years and when she had ticked a box to say that her partner was in a position of trust (eg, a teacher or youth worker) or that alcohol had affected her sexual behavior. These were chosen as factors that would trigger further questioning in a clinic environment as part of standard protocols.

A final section included questions from the young person interviewer and an opportunity for the participant to add anything else they felt relevant.

### Data Analysis

Interviews were analyzed using the framework approach [9], a matrix-based analytic method that classifies and organizes data according to key themes, subthemes, and emergent categories [10]. The interview texts were read and reread by PB and VSH before developing coding categories which were then refined through three rounds of coding and modification through discussion between PB and VSH (see Multimedia Appendix 1 for coding categories). A third researcher (JS) checked the coding of the first six transcripts (43%, 6/14) to identify initial inconsistencies in coding. As the coding categories became increasingly robust and familiar to the coders, this checking function was no longer required. Differences were resolved through discussion. The cointerviewers commented on a preliminary analysis and draft conclusions.

## Results

No respondent described young people visiting sexual health services primarily to disclose concerns about sexual exploitation. It was assumed that information about sexual exploitation would be identified during an interaction primarily focused on sexually transmitted infection or pregnancy.

Six themes emerged from the data that were grouped into two sections:

Issues relevant to the identification of CSE. The themes in this section were access, trust, and strategies for data collection. We identified two strategies that we termed “information gathering” and “information providing.”

Issues relevant to the response to CSE. The themes in this section were the consistency and effectiveness (in terms of stopping the exploitation) of responses to disclosure and online services as part of a wider system.

### Issues Relevant to the Identification of Child Sexual Exploitation

Respondents described increased access to online services with no risk of being seen using the clinic, no need to travel to the service, adapt to clinic opening hours, or wait to be seen. However, online services require private Internet access, health literacy, proficiency in English, and confidential access to mail at home.

Trust in the service was described as essential to support disclosure of CSE. The development of trust may require several visits, including testing visits. Trust includes confidence that services are nonjudgmental, will keep personal information confidential, and will reliably respond to the self-identified needs of young people at the pace they choose. In clinics, skilled clinicians may build trust quickly within the consultation and have the advantage of verbal and nonverbal cues to communicate their reliability. However, questioning can be overwhelming for young people and health professionals might lack time or skills to question appropriately. Online services provide a less pressured environment than clinics for building trust. They provide consistency with standardized questions and responses, time for young people to reflect, and a potentially anonymous space for young people to test service responses. However, online services remain faceless and it is easier to ignore key information or disengage with the process:

We know that some of the reasons that children phone Childline are because they don’t want to go in through a door. We know that some of the reasons that people contact Brook first without anybody going to see them are that they want to test that you’re okay and then they will build that trust.

Respondent 6

*If very good and clear information was put up about safeguarding, it would give young people an opportunity to really read it and think about it and be able to make decisions on that...it’s sometimes quite confrontational where you’ve got a face-to-face contact in a clinic and you’re being told and it’s a bit scary anyway, and you’re not taking it on board.* (Respondent 13)

During analysis of the theme of trust two differing approaches to safeguarding emerged: information providing and information gathering (see Textbox 2).

**Information-providing versus information-gathering approaches to identification of child sexual exploitation.**

**Information-Gathering Approach**

**Identify contextual and nonverbal clues**

“If a young person is delaying their responses or being fidgety or looks scared or concerned when a topic of conversation is raised, I think that gives professionals who have years of experience dealing with young people quite a lot of information” (Respondent 4)

**Collect information from a range of sources**

“They might not be registered with a GP...But then they might be known to lots of other agencies so that (the online service) could be a good way of tracking them down” (Respondent 13)

**Professional risk assessment**

“Then you just really have to cue into pauses and stumbling over words. And inconsistencies as well...But sometimes it doesn’t all add up...you have to be very direct in saying, ‘First of all you said this and now you’re saying that’” Respondent 8

**Young people will withhold information to prevent referral**

“Sometimes they won’t give you that detail because they know what’s going to happen with that information, so they’d rather not” (Respondent 9)

**People lie**

“Ultimately you’re fighting against people who may be intentionally deceptive” (Respondent 4)

**Information-Giving Approach**

**Creating a safe space supports disclosure:**

“If they’re...not harried and harassed, if they’re given the opportunity to actually go and access something where they feel somebody will listen to them kindly” (Respondent 13)

**Use the information given**

“So, if they don’t reveal it, they don’t reveal it. I’ve always said in safeguarding, you can only do what you do on the information you’re given and actually if you start digging around people might just go, poof and not be seen ever again” (Respondent 13)

**Young people assess their own risk**

“Where people are risk assessing themselves you’re relying on people to be seeking the level of advice that they need” (Respondent 6)

**Young people will seek help when they need it**

“If a person feels that they can trust this entity providing these services they may disclose what they are ready to disclose, if they are not ready to disclose then they will not give that information” (Respondent 11)

**People will tell the (broad) truth**

“People...will generally tell you the truth within limits, adults who go to the doctor who asks how much they drink will often say more than they’re supposed to but not quite as much as they do, by doing that enough to know a doctor’s going to say, ‘You need to worry about this a little bit’” (Respondent 6)

The information-providing approach considers that young people experiencing CSE will ask for help from someone they trust. The role of a service is to meet the criteria for young people to have sufficient trust to seek help when they need it, prompt recognition of risk by providing information, and to respond appropriately and in a timely fashion. The information-providing approach values the online service as an option that provides a safe space to test out disclosure without commitment.

The information-gathering approach considers that young people may withhold information out of concern that it might trigger an intervention or judgmental response. Therefore, services are obliged to seek out information and to form a judgment about the young person’s situation that is independent of the young person’s account. The information-gathering approach values the extended opportunities for individualized questioning to assess risk and challenge perceptions of sexual relationships and the extended contact afforded by presence in the clinic.

### Issues Relevant to the Response to Child Sexual Exploitation

When CSE is identified, a rapid response that stops the exploitation is an important outcome. Respondents noted that face-to-face services are better placed to provide immediate protection than online services. These views reference the advantages of the young person’s physical presence in the clinic at the time of disclosure that facilitates immediate involvement of social services and or the police, whereas contact online is more fragile especially when there is ambivalence about disclosure. This is particularly the case if inaccurate contact details are provided online. A dichotomy emerged in this analysis in which participants described the advantages of a user-controlled response to CSE or a provider-controlled one. Those who favored the provider-controlled response referenced the need to respond immediately:

If somebody has come out with that you have got to grab hold of it while it is there because you might not get another chance.

Respondent 14

Those who favored a user-controlled response referenced the futility of a response without user support because it was likely to lead to the retraction of the disclosure.

Participants felt strongly that to offer effective responses to the disclosure of CSE online services must be integrated into a wider clinical system. Standalone online services may offer limited knowledge of, or connections with, local support services; therefore, there are reduced opportunities for effective referral or follow-up in face-to-face services.

## Discussion

Child sexual exploitation is a subset of child sexual abuse (CSA). The nature and dynamics of CSA make it extremely difficult for young people to disclose exploitation [11]. Barriers to disclosure include dependency, strategies employed by perpetrators to maintain silence, feelings of guilt and responsibility, and fear of not being believed [11,12]. Although most CSA is first disclosed to peers and family members, approximately 10% is disclosed to professionals, including sexual health professionals [13]. Disclosure of CSE is further complicated by the young person not recognizing they are being exploited. An environment that encourages recognition of exploitation and disclosure providing a prompt response is essential to support the young person to stop the exploitation and to reduce the risk of long-term negative outcomes [11].

Online services may offer advantages in supporting the disclosure of CSE by offering consistent information, signaling an appropriate response to disclosure; facilitating initial and repeat (often testing) visits; and by providing time for reflection. Online services provide immediate, consistent, and nonjudgmental responses with a sense of safety and control that comes through remote access [14] and the possibility of “space for negotiation” rather than immediate response [15]. More sensitive information is reported via computer questionnaires than face-to-face interviews [16,17], and self-completed questionnaires can be effective in identifying CSE [18]. The “faceless” and “voiceless” nature of these services are important for young people who wish to discuss personal problems [14], particularly when these problems are stigmatizing [19]. Confidential telephone counseling services that require no identifying information are important resources for young people in crisis situations [20], such as those contemplating suicide [21].

**Figure 1:**
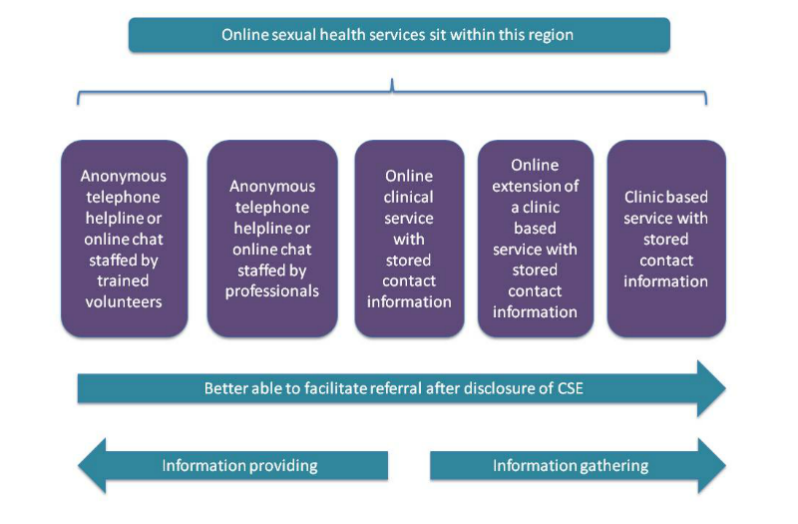
Spectrum of approaches to supporting disclosure of child sexual exploitation.

Our distinction between the information-providing and the information-gathering approaches can help further thinking about this issue. Figure 1 depicts the spectrum of remote support services for young people who are experiencing CSE. Organizations on the left of the diagram are less likely to have contact details for the young person and focus predominately on providing support until the young person is ready to disclose. Organizations on the right of the diagram are more likely to have contact details, be in a position to crosscheck information, and to insist on referrals.

Our research suggests that depending on their approach and the range of services they provide (anonymous telephone support, online chat, online clinical services that store more or less information), online services may position themselves more to the left or the right of the diagram in Figure 1 and, therefore, may adopt more of an information-gathering or an information-providing approach. The approach should be clearly signaled to users so that they are aware of the consequences of disclosure.

However, at some point, the response to disclosure of CSE will require face-to-face contact to involve the relevant services (eg, social workers and the police) and to stop the exploitation. This requires transfer of the conversation from the online service to a different context. This is the key concern about safeguarding online. We have found no published evidence about referrals between online sexual health services and clinics, although referrals from telephone helplines on all topics are successful in approximately 50% of cases [20].

From our interviews with expert practitioners, we can see a consensus that services embedded within wider clinical or organizational structures may be more effective at supporting safeguarding of children than standalone services.

Further research is needed on how to affect this transfer. This research could usefully focus on any of the following questions:

What proportion of those who disclose CSE online can be effectively referred to face-to-face services?

Is an information-gathering or an information-providing approach more effective in (1) supporting disclosure of CSE and (2) referral to face-to-face services?

Are online sexual health services more effective in supporting disclosure of CSE (whatever the approach adopted) than face-to-face services?

None of the participants had experience working in an online sexual health service, although the respondents from Brook and the Children’s charity had extensive experience providing support via telephone and online chat to young people in need of help. Participants come from a range of professional backgrounds linked to safeguarding. Some of the respondents worked almost exclusively with young people who are at risk of, or are, being sexually exploited, whereas others worked with young people seeking contraceptive or sexual health services. Young people were not included as participants in this study, although they were involved in later service development work.

In conclusion, some elements of online sexual health services may facilitate disclosure of CSE. Effective transition from online to clinic services after disclosure is an essential element of this process and further research is needed to understand how this transition can be supported.

## Appendix

Multimedia Appendix 1 Coding categories.

## Abbreviations

CSA: child sexual abuse
CSE: child sexual exploitation
