# Supplementary material for: Screening for Child Sexual Exploitation in Online Sexual Health Services: An Exploratory Study of Expert Views
Source: J Med Internet Res. 2017 Feb 14;19(2):e30. doi: 10.2196/jmir.5911 (PMC5331185; doi:10.2196/jmir.5911)
Supplement: Multimedia Appendix 1 [file jmir_v19i2e30_app1.pdf]

Appendix 1

Table 1: Final coding categories

Theme	Sub themes
Young people's beliefs and attitudes towards sex	Perceptions of normal and abnormal sexual behavior and understanding consent
Access to service for those at risk of CSA	Barriers and facilitators of access to online and clinic based services
User/provider relationships	Trust building; supporting disclosure; testing visits; transparency; power dynamics; communication; responding to a range of clinical and social needs.
Quality and consistency	Risk assessment; health promotion; assessment of competence; data governance; follow up.
Effectiveness - ability of system to identify and respond to CSA	Responding in an appropriate and timely way; identifying medical needs; identifying safeguarding concerns.
Working as part of a system	Alternative sources of support; complementarily of online and clinic based services.
Information gathering approach to CSA	Importance of face to face questioning including non-verbal clues; triangulation of information with other services; questions about accuracy of data given in this context; professional skill to identify safeguarding issues; young people's views on referral.
Information providing approach to CSA	Creating a safe space; ensuring positive response; providing information about healthy relationships; providing information about sexual health; supporting reflection; building trust; relying on young people to assess their own risk.
